# Neuroplastic changes in c‐Fos, ΔFosB, BDNF, trkB, and Arc expression in the hippocampus of male Roman rats: differential effects of sexual activity

**DOI:** 10.1002/hipo.23448

**Published:** 2022-06-18

**Authors:** Fabrizio Sanna, Maria Pina Serra, Marianna Boi, Jessica Bratzu, Laura Poddighe, Francesco Sanna, Antonella Carta, Maria Giuseppa Corda, Osvaldo Giorgi, Maria Rosaria Melis, Antonio Argiolas, Marina Quartu

**Affiliations:** ^1^ Department of Biomedical Sciences, Section of Neuroscience and Clinical Pharmacology University of Cagliari, Cittadella Universitaria di Monserrato Cagliari Italy; ^2^ Department of Biomedical Sciences, Section of Cytomorphology University of Cagliari, Cittadella Universitaria di Monserrato Cagliari Italy; ^3^ Department of Life and Environmental Sciences, Section of Pharmaceutical, Pharmacological and Nutraceutical Sciences University of Cagliari, Cittadella Universitaria di Monserrato Cagliari Italy; ^4^ Neuroscience Institute, National Research Council of Italy, Section of Cagliari Cittadella Universitaria di Monserrato Cagliari Italy

**Keywords:** Arc, BDNF, c‐Fos, hippocampus, male Roman rats, neuroplasticity, sexual experience, ΔFosB

## Abstract

Sexual activity causes differential changes in the expression of markers of neural activation (c‐Fos and ΔFosB) and neural plasticity (Arc and BDNF/trkB), as determined either by Western Blot (BDNF, trkB, Arc, and ΔFosB) or immunohistochemistry (BDNF, trkB, Arc, and c‐Fos), in the hippocampus of male Roman high (RHA) and low avoidance (RLA) rats, two psychogenetically selected rat lines that display marked differences in sexual behavior (RHA rats exhibit higher sexual motivation and better copulatory performance than RLA rats). Both methods showed (with some differences) that sexual activity modifies the expression levels of these markers in the hippocampus of Roman rats depending on: (i) the level of sexual experience, that is, changes were usually more evident in sexually naïve than in experienced rats; (ii) the hippocampal partition, that is, BDNF and Arc increased in the dorsal but tended to decrease in the ventral hippocampus; (iii) the marker considered, that is, in sexually experienced animals BDNF, c‐Fos, and Arc levels were similar to those of controls, while ΔFosB levels increased; and (iv) the rat line, that is, changes were usually larger in RHA than RLA rats. These findings resemble those of early studies in RHA and RLA rats showing that sexual activity influences the expression of these markers in the nucleus accumbens, medial prefrontal cortex, and ventral tegmental area, and show for the first time that also in the hippocampus sexual activity induces neural activation and plasticity, events that occur mainly during the first phase of the acquisition of sexual experience and depend on the genotypic/phenotypic characteristics of the animals.

## 1 INTRODUCTION

Sexual behavior is a highly instinctually and hormonally driven stereotyped behavior; however, with many other behaviors, it has also to be learned and is modulated by experience (see Pfaus et al., 2012 and references therein). Indeed, the sexual experience causes several changes in sexual behavior making copulation more efficient and increasing sexual motivation. Accordingly, (i) sexually experienced male rats show an increased preference for receptive female rats, and better performances in classical copulatory tests than sexually unexperienced (naïve) male rats and (ii) sexual experience increases the incentive value of sexual stimuli and associated cues (Everitt, 1990; Pfaus & Everitt, 1995). In addition, the sexual experience causes also neurochemical and morphological changes in several brain areas, the most studied being the ventral tegmental area (VTA)—nucleus accumbens (Acb) pathway (Beloate et al., 2016; Fiorino et al., 1997; Pfaus & Everitt, 1995) and hypothalamic areas such as the medial preoptic area (MPOA) (Dominguez & Hull, 2005) and the paraventricular nucleus of the hypothalamus (PVN) (Argiolas & Melis, 2005; Melis & Argiolas, 2021a). These changes are mediated by molecular mechanisms that play a key role not only in the behavioral modifications in sexual performance induced by sexual experience but also in the rewarding and reinforcing effects of sexual activity (Pitchers et al., 2013). Indeed, sexual experience induces changes in the pattern of the increase in dopamine release that usually occurs in the Acb, MPOA, and PVN of male rats during sexual activity and copulation (Fiorino et al., 1997; Hull et al., 1995; Meisel & Mullins, 2006; Melis et al., 2003). The increases in dopamine release occurring in the above brain areas are associated with an increased expression of the transcriptional factor c‐Fos (a marker of neuronal activation) and of ΔFosB, a truncated form of c‐Fos, in the same areas and the VTA (Biały & Kaczmarek, 1996; Bradley & Meisel, 2001; Hedges et al., 2009; Lumley & Hull, 1999; McHenry et al., 2016; Nishitani et al., 2004; Nutsch et al., 2016; Pitchers, Frohmader, et al., 2010; Veening & Coolen, 1998; Wallace et al., 2008; Witt & Insel, 1994), and MAPK phosphorylation has been observed in limbic areas (i.e., MPOA, olfactory bulb, and amygdala) of male mice after copulation (Taziaux et al., 2011). Furthermore, changes in the number of dendrites and dendritic spines in the Acb of sexually experienced male rats have been observed (Pitchers, Balfour, et al., 2010).

Recent studies have shown that the changes in the expression of the above markers of neural activation and plasticity are differentially influenced by the genotypic/phenotypic characteristics of the animals used to study the sexual behavior. Among these, the most studied are the Roman high (RHA) and low avoidance (RLA) rats (Giorgi et al., 2007, 2019) and the membrane dopamine transporter knockout (DAT‐KO) rats and their wild type (WT) and heterozygous (HET) counterparts (Leo et al., 2018). RHA and RLA rats are two rat lines originally selected for their extremely divergent responses in the active avoidance task. They show different, often opposite behavioral traits (RHA rats are active copers, impulsive and novelty seekers, and prone to intake classical drugs of abuse, while RLA rats are reactive copers that show neophobia, anxiety, hyperemotional responses like freezing to novel situations, and are prone to develop depressive‐like symptoms) (Giorgi et al., 2007, 2019). DAT‐KO rats with their WT and HET counterparts differ in their dopaminergic tone, the first having a very high tone due to the DAT absence, the second a normal tone, and the third showing a dopaminergic tone intermediate between DAT‐KO and WT rats (Leo et al., 2018). Important for this work, both Roman and DAT‐KO rat lines display significant differences in sexual behavior, RHA rats and DAT‐KO rats exhibiting higher sexual motivation and higher levels of sexual activity than RLA rats (Sanna, Corda, et al., 2014; Sanna, Piludu, et al., 2014) and HET/WT rats (Sanna et al., 2020), respectively; besides, these differences in sexual behavior appear to be secondary to the higher tone of the mesocorticolimbic dopaminergic system present not only in DAT‐KO versus HET/WT rats (Sanna et al., 2020) but also in RHA versus RLA rats (Sanna et al., 2015; Sanna, Bratzu, Piludu, et al., 2017). Moreover, the above mentioned behavioral and neurochemical differences occur concomitantly with significant differences not only in the expression of c‐Fos and ΔFosB, but also of the brain‐derived neurotrophic factor (BDNF) and its receptor tyrosine kinase B (trkB) and the activity‐regulated cytoskeleton‐associated protein (Arc) in the mesocorticolimbic system (i.e., VTA, Acb, and mPFC) after the exposition to, and direct sexual interaction with a sexually receptive female rat, not only in RHA versus RLA rats (Sanna et al., 2019) but also in DAT‐KO versus HET/WT rats (Sanna et al., 2020). These differences were bigger in sexually naïve rats (i.e., after their first sexual experience) but still present, although reduced, in sexually experienced rats (i.e., after five copulatory tests). Taken together with the differences found in the mesocorticolimbic dopamine tone, the differences in the expression of the above markers occurring in the limbic system between RHA and RLA rats, and DAT‐KO rats and HET/WT rats, led to suggest that they could be responsible, at least in part, of the differences observed in sexual behavior and, more in general, in motivated behavior that characterize the two Roman rat lines (Melis et al., 2019) and DAT‐KO rats (Cinque et al., 2018; Sanna et al., 2020).

The hippocampus is another area well known for its ability to display experience‐driven neuroplastic modifications mediating adaptive behavioral changes (Alberini & Kandel, 2014), its key role in learning and memory (Eichenbaum et al., 1992; Jarrard, 1993), response to stress (Duman et al., 1999; Floriou‐Servou et al., 2018) and motivated behavior as well (Bagot et al., 2015; Goto & Grace, 2008; LeGates et al., 2018). In the rat, this brain area, though displaying a highly conserved intrinsic organization, can be divided along its septo‐temporal axis into the dorsal (dHC) and ventral hippocampal (vHC) subregions whose functional role, due to their distinct afferent and efferent projections, varies from a preferential involvement of the dHC in the processing of spatial–temporal navigation to a preferential role of the vHC in the processing of emotional memories and related behaviors (Fanselow & Dong, 2010; Tanti et al., 2013). Nonetheless, to our knowledge, only a few studies investigated the effect of sexual activity on hippocampal neuroplasticity, revealing that sexual behavior can induce neurogenesis and is also able to stimulate the growth of dendritic spines, thus inducing a rearrangement of the hippocampal dendritic architecture (Bedos et al., 2018; Glasper & Gould, 2013; Leal‐Galicia et al., 2019; Leuner et al., 2010). However, the molecular mechanisms at the basis of these effects of sexual activity are still unknown. To obtain further information on whether sexual activity activates neuroplastic processes in the hippocampus, we measured the levels of c‐Fos, ΔFosB, BDNF, trkB, and Arc, in the dHC and vHC of control (no sexual behavior), sexually naïve (never exposed before to sexual stimuli—after the first copulatory test) and experienced (i.e., after five copulatory tests) male RHA and RLA rats using Western Blot and/or immunohistochemistry. We then verified whether the levels and changes of these markers were related to the well‐known differences in the acquisition of sexual experience, and in sexual motivation and copulatory performance displayed by the two Roman lines (Sanna, Corda, et al., 2014; Sanna, Piludu, et al., 2014).

Since RHA and RLA rats were expected to display the already known differences in copulatory behavior (RHA rats display a more rapid acquisition of sexual experience, e.g., a stable level of sexual activity, and higher levels of sexual motivation and performance, i.e., shorter latencies to mount, intromit and ejaculate, and a higher intromission ratio and ejaculatory frequency than RLA rats), we hypothesized that: (i) these sexual differences should occur concomitantly with line‐related differences in the expression of one or more of the above markers in the dHC and/or vHC (e.g., RHA should be expected to display greater experience‐induced differences than RLA rats and, in particular, higher levels of c‐Fos, ΔFosB, BDNF, and Arc), and (ii) naïve rats of both Roman lines should display greater changes compared to the experienced ones (Sanna et al., 2019).

## 2 MATERIAL AND METHODS

### 2.1 Animals

Outbred male RHA and RLA rats (*n* = 45 for each rat line) weighing ≈300 g at the beginning of the experiments were from the colony established in 1998 at the University of Cagliari, Italy (Giorgi et al., 2007). Details on the procedures used for the selective breeding of the Sardinian colony are in Giorgi et al. (2005). Ovariectomized Sprague Dawley female rats (250–300 g) were from Envigo (San Pietro al Natisone, Italy). Upon arrival, rats underwent habituation to the housing facilities of the Department of Biomedical Sciences of the University of Cagliari for at least 10 days before the beginning of the experiments. During the experimental period, they were kept 4 per cage (38 cm × 60 cm × 20 cm) under controlled environmental conditions (22 ± 2°C, 60% humidity, reversed 12 h light/dark cycle, with lights off from 07:00 to 19:00 h) and with tap water and standard laboratory food ad libitum. To minimize stress due to the experimental procedures each rat was daily handled for approximately 1–2 min throughout the habituation period, contact with the animal house maintenance personnel was limited to a single attendant, and bedding in the home cages was never changed either the day before or on the day of the experiment. All the experiments took place between 10:00 and 18:00 h. This study conformed with the guidelines of the European Communities, Directive of September 22, 2010 (2010/63/EU) and the Italian Legislation (D.L. 4, 2014, n. 26). The experimental protocol was approved by the Committee for Animal Experimentation of the University of Cagliari and authorized by the Italian Ministry of Health (Authorization No. 361/2016‐PR, April 8, 2016, to FS).

### 2.2 Experimental groups

Sexually naïve male RHA and RLA rats (*n* = 15 for each line) were rats never exposed before the experiment to a sexually receptive female rat and/or sexual stimuli. Sexually experienced male RHA and RLA rats (*n* = 15 for each line) were rats that underwent five consecutive 60 min copulatory tests at 3‐day intervals (Sanna et al., 2015, 2019; Sanna, Bratzu, Piludu, et al., 2017; Sanna, Corda, et al., 2014; Sanna, Piludu, et al., 2014). Briefly, male rats from each line were moved during the dark phase of the cycle to a soundproof room lit by a dim red light where they were housed individually in a mating cage (45 cm × 30 cm × 24 cm) for 30 min. At the end of this habituation period, a stimulus female rat was introduced into the mating cage, and sexual interaction was allowed for 60 min. Each male rat received a different stimulus female rat in every test. Oestrus was induced in ovariectomized female rats by subcutaneous injections of estradiol benzoate (200 μg/rat in 0.2 ml of peanut oil) and progesterone (0.5 mg/rat in 0.2 ml of peanut oil) 48 and 6 h before the behavioral tests, respectively and the oestrus condition was ascertained 1 h before the tests by microscopical examination of May‐Grünwald Giemsa stained vaginal smears (Contini et al., 2018). In agreement with our previous studies (Sanna et al., 2015, 2019; Sanna, Bratzu, Piludu, et al., 2017; Sanna, Corda, et al., 2014; Sanna, Piludu, et al., 2014), after five preliminary tests, rats displayed stable levels of copulatory activity with a receptive female rat (e.g., they become sexually experienced). To determine the basal expression of the molecular markers by Western Blot and immunohistochemistry, age‐matched rats of both RHA and RLA lines (*n* = 15 for each line) never exposed to sexual stimuli and/or copulatory tests were used as controls (see below).

### 2.3 Sexual behavior

**FIGURE 1:**
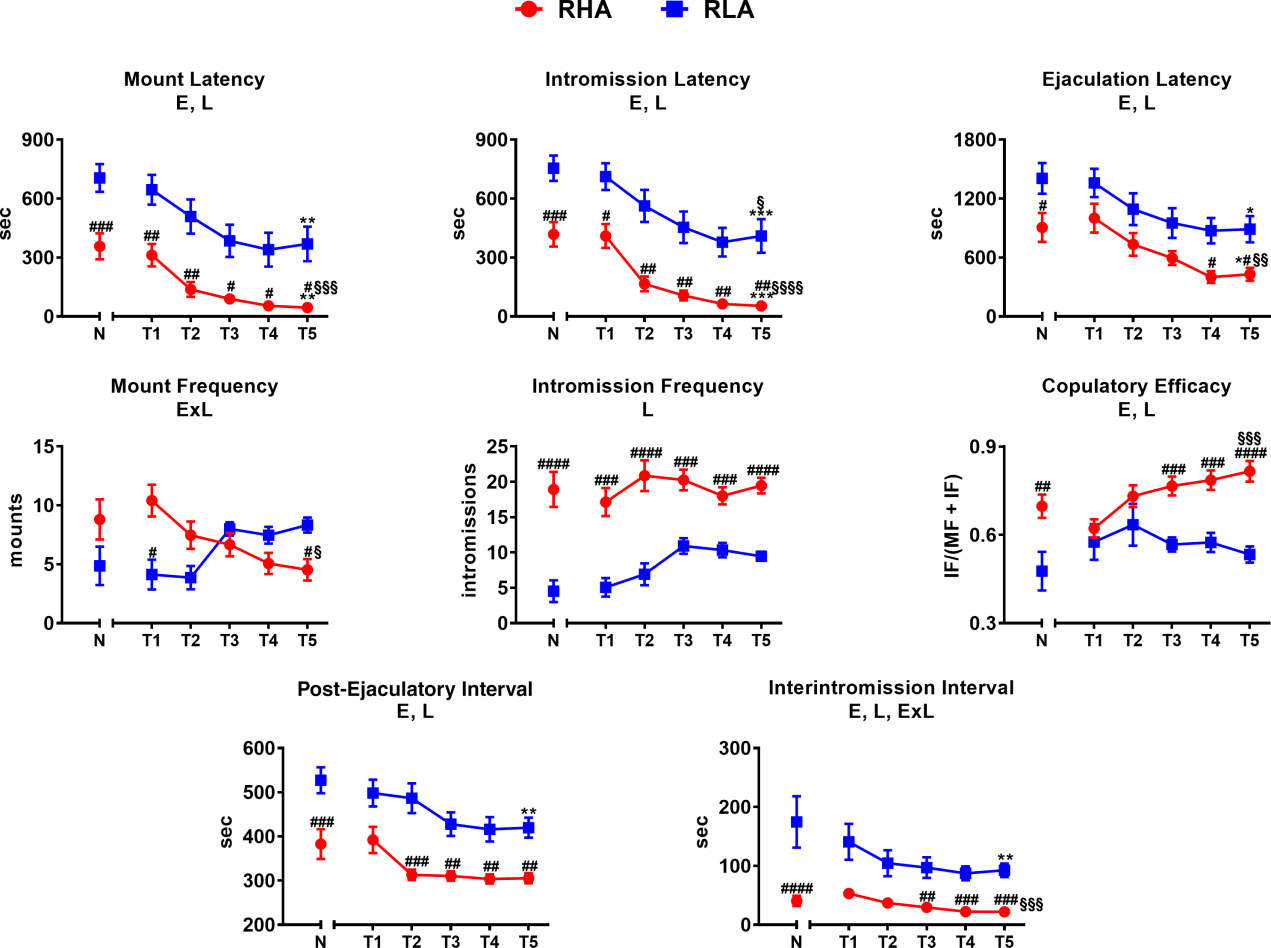
Sexual behavior of male experienced RHA and RLA rats in the first series of five copulatory tests (T1–T5). Rats from naïve groups (N) are reported for comparison. For a detailed description of the experimental procedures and the parameters of sexual behavior see Section  2.3. Values are means ± *SEM* of 15 rats per group. Two‐way ANOVA followed by pairwise comparisons. E, significant effect of level of sexual experience; L, significant effect of line; E × L, significant interaction of E and L. #, ##, ###, #### = *p* < .05, .01, .001, .0001, RHA versus RLA; *, **, *** = *p* < .05, .01, .001, experienced versus Naïve; §, §§, §§§, §§§§ = *p* < .05, .01, .001, .0001, T5 versus T1

**FIGURE 2:**
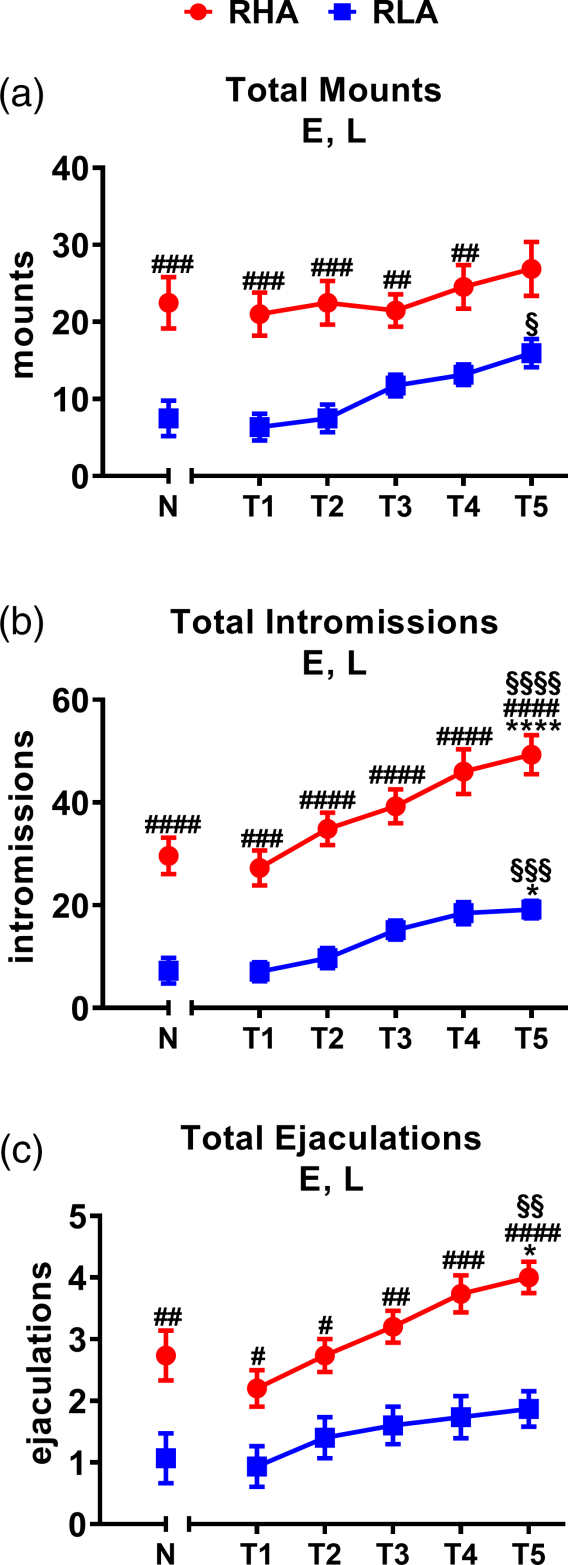
Total number of mounts (a), intromissions (b), and ejaculations (c) performed by male experienced RHA and RLA rats during the entire 60 min of five copulatory tests (T1–T5). Rats from naïve groups (N) are reported for comparison. The experimental conditions are identical to those of data reported in Figure 1. Values are means ± *SEM* of 15 rats per group. Two‐way ANOVA followed by pairwise comparisons. E, significant effect of level of sexual experience; L, significant effect of line. #, ##, ###, #### = *p* < .05, .01, .001, .0001, RHA versus RLA; *, **** = *p* < .05, .0001, experienced versus Naïve; §§, §§§, §§§§ = *p* < .01, .001, .0001, T5 versus T1

During the copulatory tests, several parameters of sexual motivation and copulatory performance were recorded by an observer who was not aware of the experimental conditions (i.e., who was not aware of the line and the level of sexual experience of the animals). Briefly, the following parameters referred to the first series of copulatory activity (timed from the first mount/intromission after the introduction of the female to the first mount/intromission after the first ejaculation) were recorded and reported in Figure 1: mount and intromission latency (ML and IL, the time elapsed since the introduction of the female into the mating cage until the first mount or the first intromission, respectively); mount and intromission frequency (MF and IF, the number of mounts and intromissions in the first series of copulatory activity, respectively); ejaculation latency (EL, timed from the first intromission until the first ejaculation), and post‐ejaculatory interval (PEI, timed from the first ejaculation until the next mount/intromission). In addition, the following parameters were also calculated: copulatory efficacy (CE, the number of intromissions of the first series divided by the sum of the number of mounts and of intromissions in that series) and inter‐intromission interval (III) (the ratio between the EL of the first series and the IF in that series). Finally, the total number of mounts (TMF), intromissions (TIF), and ejaculations (TEF) recorded during the whole 60 min test were also recorded and reported in Figure 2 (Le Moëne & Ågmo, 2019; Sachs & Barfield, 1976; Sanna et al., 2019; Sanna, Corda, et al., 2014; Sanna, Piludu, et al., 2014; and references therein).

### 2.4 Tissue sampling

Ninety to 120 min after the end of the copulatory tests (e.g., after the first or the fifth copulatory test for sexually naïve and experienced rats, respectively), male rats were deeply anesthetized with intraperitoneal Equitesin (Pentobarbital 0.97 g, MgSO_4_ 2.1 g, chloral hydrate 4.25 g, propylene glycol 42.8 ml, ethanol 90% 11.5 ml, to 100 ml with sterile distilled H_2_O; 0,5 ml/kg body weight) and were either decapitated and brains rapidly removed and processed for Western Blot or transcardially perfused with ice‐cold 0.1 M phosphate buffer (PB), pH 7.3 for immunohistochemistry. Control rats were sacrificed at the same time points as sexually naïve and experienced rats. For Western Blot, brains were cooled in dry ice for 15 s, placed in a brain matrix, and cut into 2 mm thick coronal slices using the stereotaxic coordinates of the rat brain atlas of Paxinos and Watson (2004) as a reference. The AP coordinates (from Bregma) were approximately −3.30 mm for the dHC and −6.04 mm for the vHC. Bilateral punches (diameter 2.5 mm) of the dHC and vHC were taken, as described by Palkovits (1983). For each rat, the tissue punches from both hemispheres were pooled, rapidly frozen at −80°C, and homogenized in distilled water containing 2% sodium dodecylsulfate (SDS) (300 μl/100 mg of tissue) and a cocktail of protease inhibitors (cOmpleteTM, Mini Protease Inhibitor Cocktail Tablets, Cat# 11697498001, Roche, Basel, Switzerland). For immunohistochemistry, the perfused brains were fixed by overnight immersion in freshly prepared 4% phosphate‐buffered formaldehyde, pH 7.3, at 4°C, as previously described (Quartu et al., 2010; Serra et al., 2017), and then rinsed until they sank in 0.1 M PB, pH 7.3, containing 20% sucrose.

### 2.5 Western blot

Determination of total protein concentrations was carried out according to Lowry's method (Lowry et al., 1951). Proteins from each tissue homogenate (40 μg), diluted 3:1 in 4× loading buffer (NuPAGE LDS Sample Buffer 4×, Cat# NP0008, Novex by Life Technologies, Carlsbad, CA, USA), were then heated to 95°C for 7 min and separated by SDS‐polyacrylamide gel electrophoresis (SDS‐PAGE) using precast polyacrylamide gradient gel (NuPAGE 4%–12% Bis‐Tris Gel Midi, Cat# NP0321, Novex by Life Technologies) in the XCell4 Sure LockTM Midi‐Cell chamber (Life Technologies). Internal molecular weight (MW) standards (Precision Plus Protein Western C Standards, Cat# 161‐0376, Bio‐Rad, Hercules, CA, USA) were run in parallel. Blots were blocked by immersion in 20 mM Tris base and 137 mM sodium chloride (TBS) containing 5% milk powder and 0.1% Tween 20 (TBS‐T), for 60 min at room temperature. Rabbit monoclonal antibody against ΔFosB (Cat#14695; RRID:AB_2798577, Cell Signaling Technology), diluted 1:1000, rabbit polyclonal antisera against BDNF (Cat# N‐20 sc‐546, RRID:AB_630940, Santa Cruz Biotechnology), diluted 1:500, and trkB (Cat# (794) sc‐12, RRID:AB_632557, Santa Cruz Biotechnology) diluted 1:1000, and mouse monoclonal antibody against Arc (Cat# sc‐17839, SCBT, Santa Cruz, CA, USA), diluted 1:300, in TBS containing 5% milk powder and 0.02% sodium azide, were used as the primary antibody. Equal loading of the wells was checked by using a mouse monoclonal antibody directed against glyceraldehyde‐3‐phosphate dehydrogenase (GAPDH) (MAB374, RRID:AB_2107445, EMD Millipore, Darmstadt, Germany), diluted 1:1000, as the primary antiserum. Incubations with primary antibodies were performed at 4°C and lasted 24 h for Arc and GAPDH, two nights for BDNF and trkB, and five nights for ΔFosB. Blots were then rinsed in TBS/T, incubated for 60 min, at room temperature, with peroxidase‐conjugated goat anti‐rabbit serum (Cat#9169, RRID:AB_258434, Sigma Aldrich, St Louis, MO, USA), diluted 1:10,000, and goat anti‐mouse serum (AP124P, RRID:AB_90456, Millipore, Darmstadt, Germany), diluted 1:5000 in TBS/T, as the secondary antiserum. In order to control for nonspecific staining, blots were stripped and incubated with the relevant secondary antiserum. After TBS/T rinse, protein bands were developed using the Western Lightning Plus ECL (Cat# 103001EA, PerkinElmer, Waltham, MA, USA), according to the protocol provided by the company, and visualized by means of ImageQuant LAS‐4000 (GE Healthcare, Little Chalfont, UK). Approximate MW and relative optical density (OD) of labeled protein bands were evaluated by a blinded examiner. The ratio of the intensity of ΔFosB‐, BDNF‐, trkB‐ and Arc‐positive bands to the intensity of GAPDH‐positive ones was used to compare relative expression levels of these proteins in the RHA and RLA lines. Image Studio Lite Software (RRID:SCR_014211, Li‐Cor, http://www.licor.com/bio/products/software/image_studio_lite/) was used to quantify the OD of each sample.

### 2.6 Immunohistochemistry

Coronal sections of RLA and RHA rat brains were examined in pairs on the same slide. Sections (14 μm thick) were cut with a cryostat and collected on chrome alum‐gelatin coated slides and processed by the avidin–biotin–peroxidase complex (ABC) immunohistochemical technique. The endogenous peroxidase activity was inhibited with 0.1% phenylhydrazine (Cat# 101326606, Sigma Aldrich, St Louis, MO, USA) in phosphate‐buffered saline (PBS) containing 0.2% Triton X‐100 (PBS/T), followed by incubation with 20% of normal goat serum (Cat# S‐1000, RRID:AB_2336615, Vector Labs Inc., Burlingame, CA, USA). The same rabbit polyclonal antibodies used for Western Blot, that is, antibodies against BDNF (Santa Cruz Biotechnology, Santa Cruz, CA, USA), diluted 1:400, and trkB (Santa Cruz Biotechnology, Santa Cruz, CA, USA), diluted 1:500, a sheep polyclonal anti‐c‐Fos antibody (Cat# sc‐52, RRID:AB_2106783; Santa Cruz Biotechnology, Santa Cruz, CA, USA), diluted 1:1000, and a rabbit polyclonal antibody against Arc (Cat# sc‐15325, RRID:AB_634092; Santa Cruz Biotechnology, Santa Cruz, CA, USA), diluted 1:1000, were used as primary antibody. Biotin‐conjugated goat anti‐rabbit (Cat# BA‐1000, RRID:AB_2313606 Vector Labs Inc., Burlingame, CA, USA) and anti‐sheep (Cat# BA‐6000, RRID:AB_2336217, Vector Labs Inc., Burlingame, CA, USA), both diluted 1:400, were used as secondary antiserum. The reaction product was revealed with the ABC (Cat#G011‐61, BioSpa Div. Milan, Italy), diluted 1:250, followed by incubation with a solution of 0.1 M PB, pH 7.3, containing 0.05% 3,3′‐diaminobenzidine (Sigma Aldrich, St Louis, MO, USA), 0.04% nickel ammonium sulfate and 0.01% hydrogen peroxide. All antisera and the ABC were diluted in PBS/T. Incubation with primary antibodies was carried out at 4°C and lasted 18 h for c‐Fos and trkB and 66 h for BDNF and Arc. Incubations with secondary antiserum and ABC lasted 60 and 40 min, respectively, and were performed at room temperature. Negative control preparations were obtained by incubating tissue sections in parallel with either PBS/T alone or, as in the case of the antibodies against BDNF and trkB, with the relevant primary antiserum preabsorbed with an excess of the corresponding peptide antigen (Cat# sc‐546P and sc‐12 P, for BDNF and trkB, respectively, Santa Cruz Biotechnology, Santa Cruz, CA, USA). Slides were observed with an Olympus BX61 microscope connected to a Leica DFC450C camera for the digital acquisition of images.

### 2.7 Image densitometry

For the quantitative evaluation of BDNF, trkB, c‐Fos, and Arc immunohistochemical labeling, 10× magnification representative microscopic fields, taken from six animals for each experimental group, scoring 12 different microscopic fields per brain region, were blindly analyzed with ImageJ (http://rsb.info.nih.gov/ij/; RRID:SCR_003070) to calculate the density of immunoreactivity per μm^2^. Mean gray values from unstained areas were subtracted from the gray values of the immunostained regions to exclude background staining.

### 2.8 Statistical analyses

Statistical analyses of molecular (BDNF, trkB, c‐Fos, ΔFosB, and Arc) and behavioral (ML, IL, EL, MF, IF, TMF, TIF, TEF, and PEI) data were conducted in each experimental subject, including those animals which did not display mounts, intromissions or ejaculations. This was done in the attempt to better ascertain whether the differences in sexual behavior between the Roman lines may correlate with differences in the expression of the molecular markers under investigation during the acquisition of sexual experience. Briefly, rats received the full range scores of 900 s if they did not display mounts and/or intromissions with the available female within the first 15 min from the begin of the test; 1800 s if they did not reach ejaculation within 30 min after the first intromission and 600 s if they did not mount/intromit within 10 min after the first ejaculation (Sanna et al., 2015, 2019, 2020; Sanna, Bratzu, Piludu, et al., 2017; Sanna, Corda, et al., 2014; Sanna, Piludu, et al., 2014).

Behavioral data obtained from naïve and sexually experienced male RHA and RLA rats during the first series of copulatory activity (i.e., from the first mount/intromission to the first mount/intromission after the first ejaculation, see Figure 1) or during the whole 60 min experiment (see Figure 2), were analyzed by two‐way analyses of variance (ANOVAs) (between‐subjects factors: rat line, level of sexual experience; within‐subjects factor: test) (see Table 1).

**TABLE 1:** Effect of sexual experience and rat line on parameters of sexual behavior

Copulatory parameters	Level of sexual experience		Line		Experience × line		
	*F* value	*p* value	*F* value	*p* value	*F* value	*p* value	*df*
Mount latency (s)	24.23	<.000	26.01	<.000	0.03	ns	1, 1, 1, 56
Intromission latency (s)	32.40	<.000	30.86	<.000	0.02	ns	1, 1, 1, 56
Ejaculation latency (s)	14.56	.0003	13.49	.0005	0.03	ns	1, 1, 1, 56
Mount frequency	0.09	ns	0.00	ns	8.80	.004	1, 1, 1, 56
Intromission frequency	2.94	ns	58.58	<.000	1.90	ns	1, 1, 1, 56
Post‐ejaculatory interval (s)	12.78	.0007	25.10	<.000	0.33	ns	1, 1, 1, 56
Copulatory efficacy	4.72	.035	38.86	<.000	0.59	ns	1, 1, 1, 46
Inter intromission interval (s)	44.72	<.000	10.80	.002	4.29	.044	1, 1, 1, 46
Total mounts	5.13	.027	20.84	<.000	0.51	ns	1, 1, 1, 56
Total intromissions	27.32	<.000	74.99	<.000	1.66	ns	1, 1, 1, 56
Total ejaculations	8.83	.004	29.85	<.000	0.45	ns	1, 1, 1, 56

*Note*: *F* values and significance levels of two‐way ANOVAs performed on data reported in Figures 1 and 2.

Abbreviation: ns, not significant.

**FIGURE 3:**
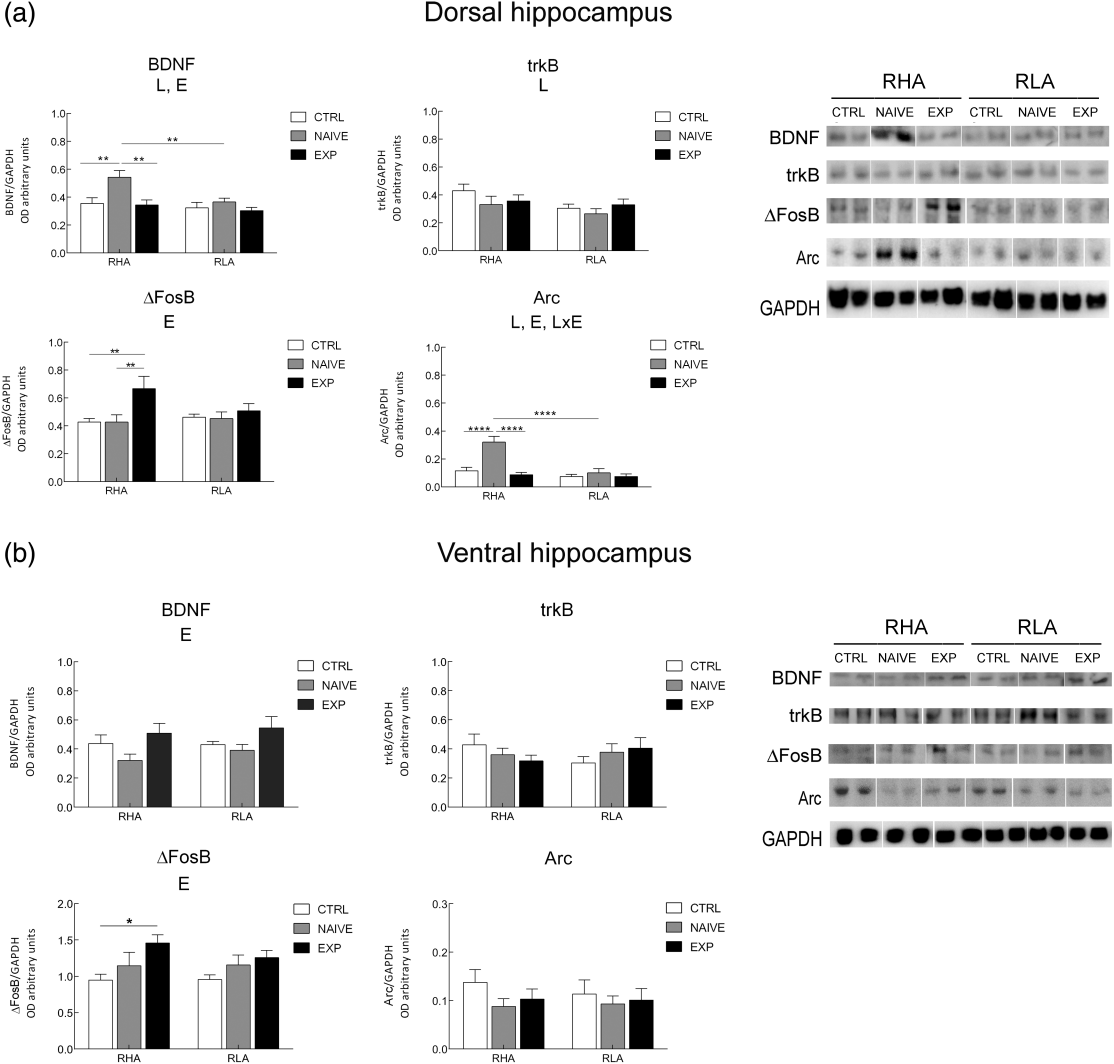
Effect of sexual activity on protein relative levels. Western blot analysis of BDNF, trkB, ΔFosB, and Arc in the dorsal (a) and ventral (b) hippocampus, of male RHA and RLA rats under control (Ctrl) (no copulation), sexually naïve (Naïve) (after the first copulatory test), and experienced (Exp) (after five copulatory tests) conditions. Shown (right) are representative samples of each experimental group and (left) densitometric analysis of the marker/GAPDH band gray optical density (OD) ratios. Values are means ± *SEM* of eight rats in each experimental group. Two‐way ANOVA followed by pairwise comparisons. L, significant effect of line; E, significant effect of level of sexual experience; E × L, significant interaction of E and L. *p < .05; **p < .01; ****p < .0001

**FIGURE 4:**
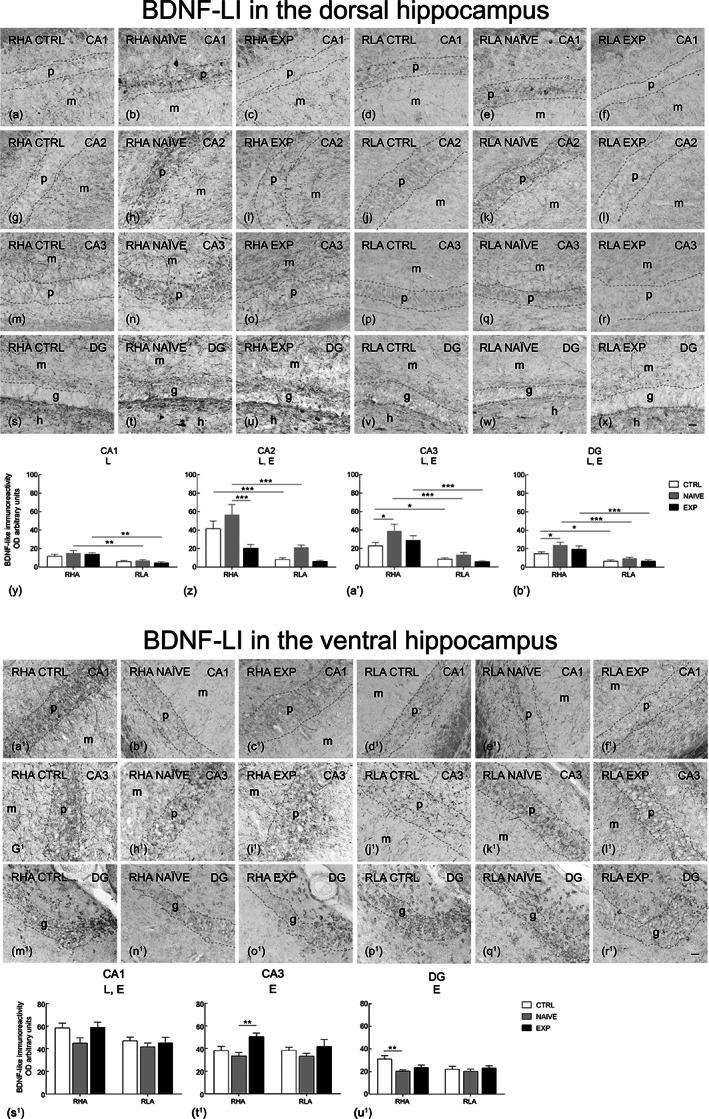
Regional distribution (a–x, a^1^–r^1^) and densitometric analysis (y–b′, s^1^–u^1^) of changes induced by sexual activity in the BDNF‐like immunoreactivity (LI) in coronal sections of the dorsal and ventral hippocampus of male RHA and RLA rats under control (Ctrl) (no copulation), sexually naïve (Naïve) (after the first copulatory test), and experienced (Exp) (after five copulatory tests) conditions. (a–f, a^1^–f^1^) CA1 sector; (g–l) CA2 sector; (m–r, g^1^–l^1^) CA3 sector of the Ammon's horn; (s–x, m^1^–r^1^) dentate gyrus (DG). Dashed lines mark the boundaries of pyramidal (p) and granular (g) layers. (y–b′, s^1^–u^1^) Densitometric analysis; values are mean ± *SEM* of six rats for each experimental group obtained by averaging the optical density (OD) from 12 different microscopic fields for each brain region. Two‐way ANOVAs followed by pairwise comparisons. E, significant effect of level of sexual experience; L, significant effect of line. *p < .05; **p < .01; ***p < .001. h, hilus; m, molecular layer. Scale bars:  a‐x = a^1^‐r^1^= 50 μm

**FIGURE 5:**
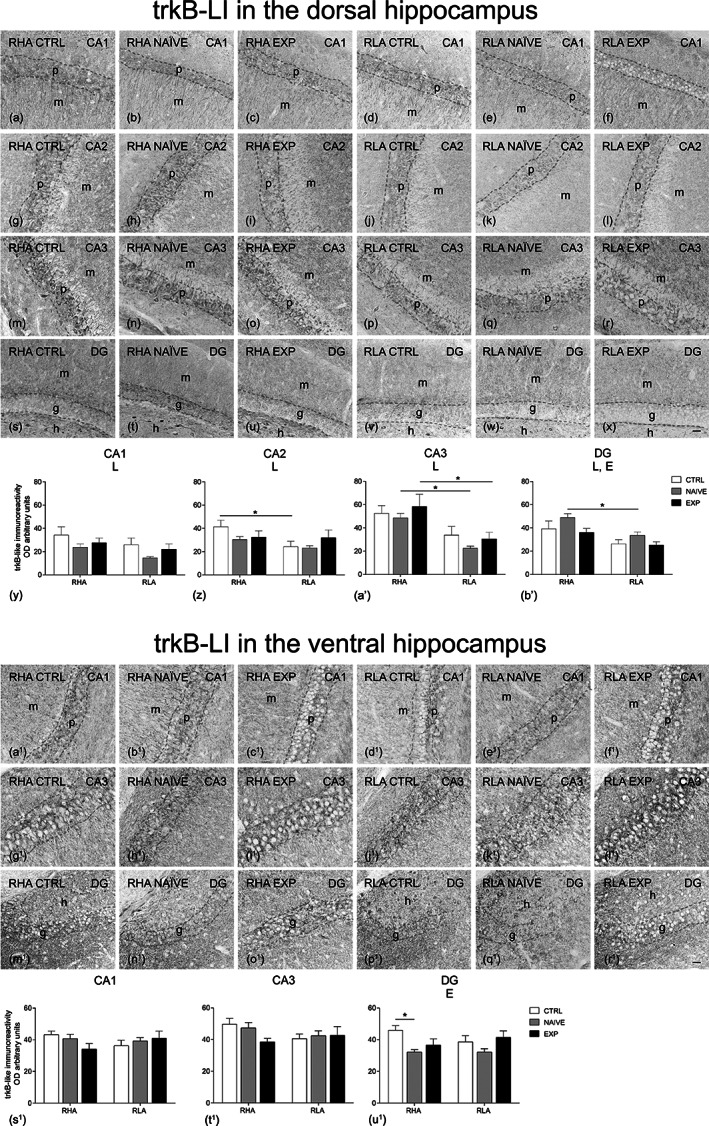
Regional distribution (a–x, a^1^–r^1^) and densitometric analysis (y–b′, s^1^–u^1^) of changes induced by sexual activity in the trkB‐like immunoreactivity (LI) in coronal sections of the dorsal and ventral hippocampus of male RHA and RLA rats under control (Ctrl) (no copulation), sexually naïve (Naïve) (after the first copulatory test), and experienced (Exp) (after five copulatory tests) conditions. (a–f, a^1^–f^1^) CA1 sector; (g–l) CA2 sector; (m–r, g^1^–l^1^) CA3 sector of the Ammon's horn; (s–x, m^1^–r^1^) dentate gyrus (DG). Dashed lines mark the boundaries of pyramidal (p) and granular (g) layers. (y–b′, s^1^–u^1^) Densitometric analysis; values are mean ± *SEM* of six rats for each experimental group obtained by averaging the optical density (OD) from 12 different microscopic fields for each brain region. Two‐way ANOVA followed by pairwise comparisons. E, significant effect of level of sexual experience; L, significant effect of line. *p < .05; **p < .01; ***p < .001. h, hilus; m, molecular layer. Scale bars: a‐x = a^1^‐r^1^ = 50 μm

**FIGURE 6:**
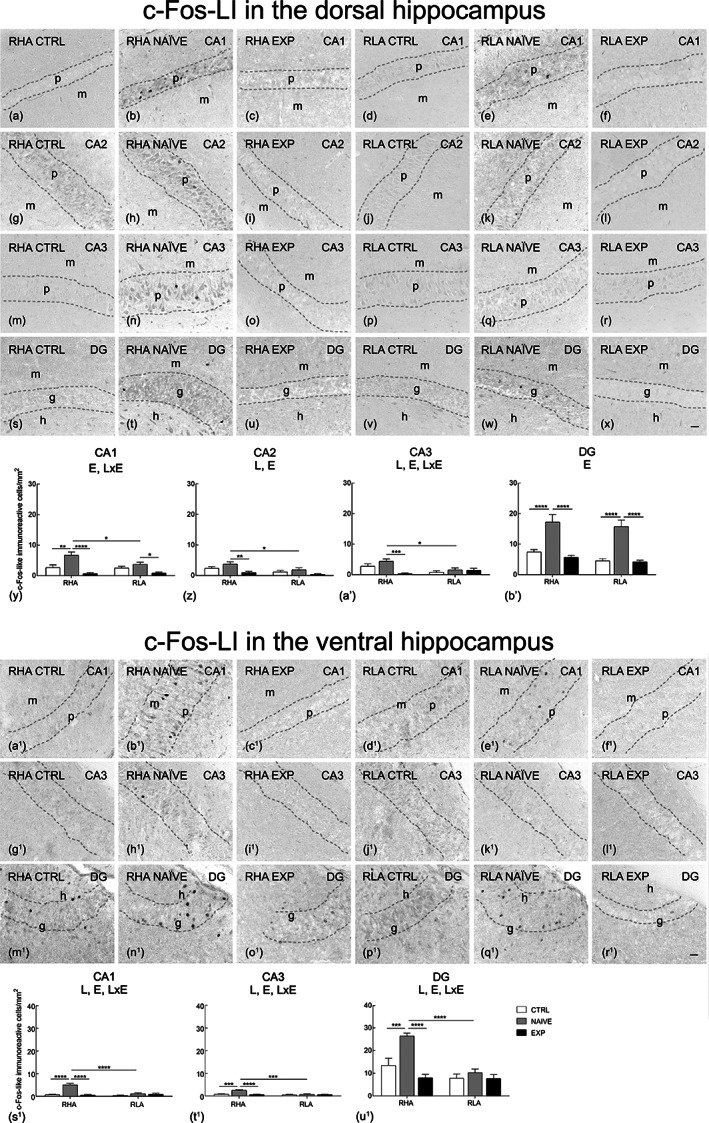
Regional distribution (a–x, a^1^–r^1^) and densitometric analysis (y–b′, s^1^–u^1^) of changes induced by sexual activity in the c‐Fos‐like immunoreactivity (LI) in coronal sections of the dorsal and ventral hippocampus of male RHA and RLA rats under control (Ctrl) (no copulation), sexually naïve (Naïve) (after the first copulatory test), and experienced (Exp) (after five copulatory tests) conditions. (a–f, a^1^–f^1^) CA1 sector; (g–l) CA2 sector; (m–r, g^1^–l^1^) CA3 sector of the Ammon's horn; (s–x, m^1^–r^1^) dentate gyrus (DG). Dashed lines mark the boundaries of pyramidal (p) and granular (g) layers. (y–b′, s^1^–u^1^) Densitometric analysis; values are mean ± *SEM* of six rats for each experimental group obtained by averaging the optical density (OD) from 12 different microscopic fields for each brain region. Two‐way ANOVA followed by pairwise comparisons. E, significant effect of level of sexual experience; L, significant effect of line. *p < .05; **p < .01; ***p < .001. h, hilus; m, molecular layer. Scale bars: a‐x = a^1^‐r^1^ = 50 μm

**FIGURE 7:**
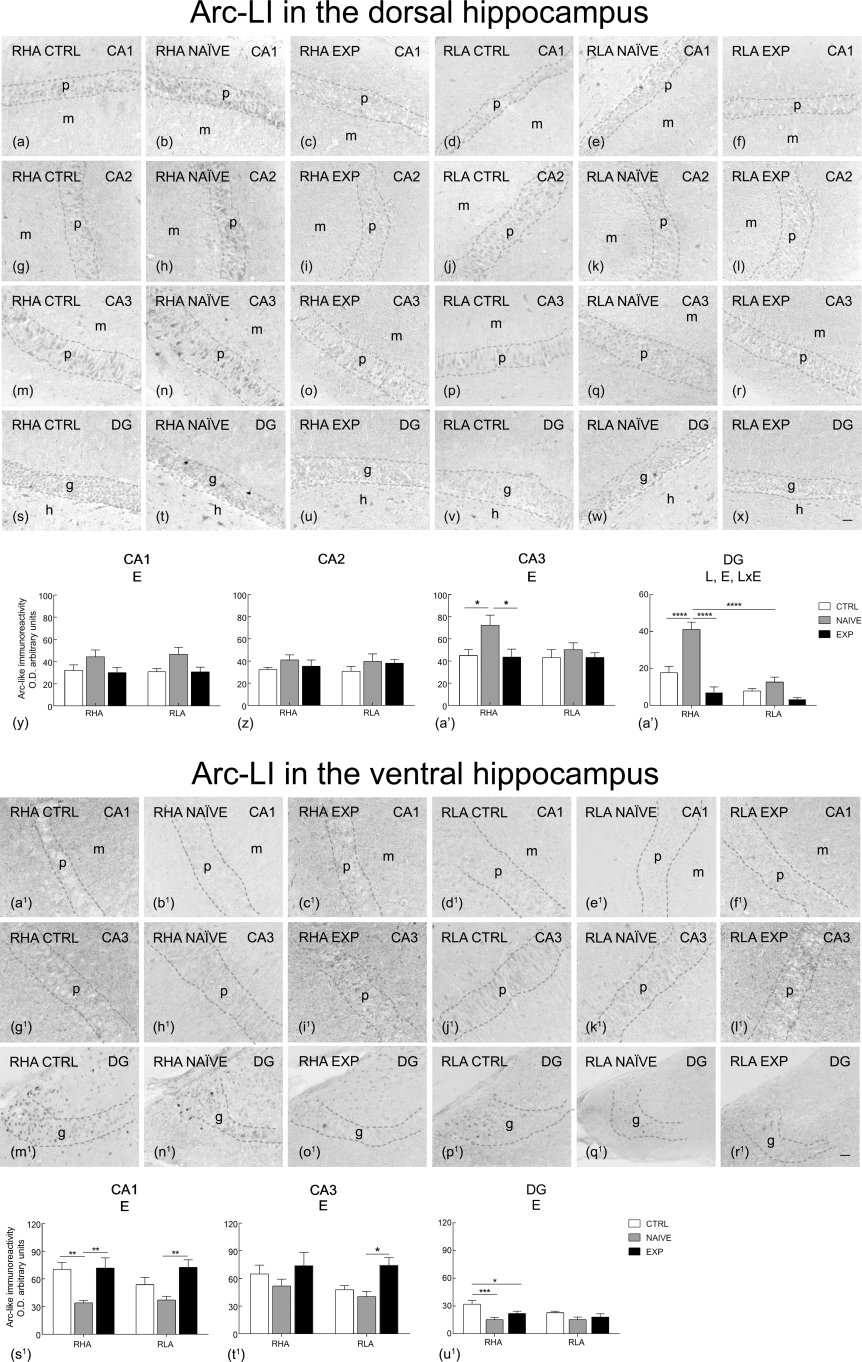
Regional distribution (a–x, a^1^–r^1^) and densitometric analysis (y–b′, s^1^–u^1^) of changes induced by sexual activity in the Arc‐like immunoreactivity (LI) in coronal sections of the dorsal and ventral hippocampus of male RHA and RLA rats under control (Ctrl) (no copulation), sexually naïve (Naïve) (after the first copulatory test), and experienced (Exp) (after five copulatory tests) conditions. (a–f, a^1^–f^1^) CA1 sector; (g–l) CA2 sector; (m–r, g^1^–l^1^) CA3 sector of the Ammon's horn; (s–x, m^1^–r^1^) dentate gyrus (DG). Dashed lines mark the boundaries of pyramidal (p) and granular (g) layers. (y–b′, s^1^–u^1^) Densitometric analysis; values are mean ± *SEM* of six rats for each experimental group obtained by averaging the optical density (OD) from 12 different microscopic fields for each brain region. Two‐way ANOVA followed by pairwise comparisons. E, significant effect of level of sexual experience; L, significant effect of line. *p < .05; **p < .01; ***p < .001. h, hilus; m, molecular layer. Scale bars: a‐x = a^1^‐r^1^ = 50 μm

The data from the densitometric analyses of the immunochemical labeling for markers investigated (see Figures 3, 4, 5, 6, 7) were also analyzed by two‐way ANOVAs (between‐subjects factors: rat line, level of sexual experience) (see Table 2 and Table 3). However, in this case, the data obtained from a control group (i.e., a group of rats never exposed to sexual stimuli and/or copulation) were added.

**TABLE 2:** Effect of sexual experience on Roman high and low avoidance rat lines

Brain area	Marker	Level of sexual experience		Line		Experience × line	
		*F* value	*p* value	*F* value	*p* value	*F* value	*p* value
dHC	BDNF	7.48	.002	7.68	.008	2.50	ns
	trkB	1.30	ns	4.14	.048	0.65	ns
	ΔFosB	5.03	.011	0.57	ns	2.15	ns
	Arc	15.39	<.000	18.87	<.000	9.62	<.000
vHC	BDNF	4.71	.014	0.51	ns	0.23	ns
	trkB	0.01	ns	0.02	ns	1.81	ns
	ΔFosB	5.76	.006	0.39	ns	0.51	ns
	Arc	1.26	ns	0.14	ns	0.22	ns

*Note*: *F* values and significance levels from two‐way ANOVAs (*df* = 2, 1, 2, 42) performed on the relative BDNF, trkB, ΔFosB, and Arc protein levels found in control (no copulation), sexually naïve (after one copulatory test) and sexually experienced (after five copulatory tests) male rats by means of Western blot analysis from the experiments shown in Figure 3.

Abbreviations: dHC, dorsal hippocampus; ns, not significant; vHC, ventral hippocampus.

**TABLE 3:** Effect of sexual experience on Roman high and low avoidance rat lines

Brain area		Marker	Level of sexual experience		Line		Experience × line	
			*F* value	*p* value	*F* value	*p* value	*F* value	*p* value
dHC	DG	BDNF	3.54	.035	38.94	<.000	0.94	ns
		trkB	3.88	.025	15.70	<.000	0.14	ns
		c‐Fos	39.34	<.000	2.78	ns	0.17	ns
		Arc	33.03	<.000	40.20	<.000	11.22	<.000
	CA1	BDNF	0.82	ns	26.85	<.000	0.49	ns
		trkB	2.76	ns	4.05	.048	0.09	ns
		c‐Fos	21.40	<.000	3.05	ns	3.20	.047
		Arc	5.68	.005	0.02	ns	0.06	ns
	CA2	BDNF	8.91	<.000	30.94	<.000	1.84	ns
		trkB	0.99	ns	4.51	.037	1.53	ns
		c‐Fos	7.99	<.000	7.82	.006	0.70	ns
		Arc	1.76	ns	0.00	ns	0.14	ns
	CA3	BDNF	3.50	.036	38.76	<.000	1.00	ns
		trkB	1.04	ns	20.12	<.000	0.27	ns
		c‐Fos	5.44	.006	5.57	.021	5.35	.007
		Arc	4.61	.013	2.16	ns	1.64	ns
vHC	DG	BDNF	4.10	.021	3.15	ns	2.64	ns
		trkB	5.00	.009	0.09	ns	1.82	ns
		c‐Fos	15.07	<.000	20.77	<.000	8.27	<.000
		Arc	9.05	<.000	3.40	ns	1.31	ns
	CA1	BDNF	3.25	.045	7.88	<.006	0.85	ns
		trkB	0.36	ns	0.04	ns	2.48	ns
		c‐Fos	22.08	<.000	13.07	<.000	14.10	<.000
		Arc	13.36	<.000	0.50	ns	1.08	ns
	CA3	BDNF	5.91	.004	0.91	ns	0.95	ns
		trkB	1.00	ns	1.20	ns	1.69	ns
		c‐Fos	8.63	<.000	9.31	.003	6.73	.002
		Arc	5.27	.007	1.72	ns	0.52	ns

*Note*: *F* values and significance levels from two‐way ANOVAs (*df* = 2, 1, 2, 66) performed on the relative densitometric units of BDNF, trkB, c‐Fos, and Arc observed in control (no copulation), sexually naïve (after one copulatory test) and sexually experienced (after five copulatory tests) male rats by means of immunohistochemistry from the experiments shown in Figures 4, 5, 6, 7.

Abbreviations: dHC, dorsal hippocampus; ns, not significant; vHC, ventral hippocampus.

Before performing ANOVAs, data sets of each experimental variable were checked for normal distribution with the Shapiro–Wilk's test and for homogeneity of variances with the Brown‐Forsythe test. When significant differences in the variances of a data set were found, ANOVA was carried out with the Geisser–Greenhouse correction. When two‐way ANOVAs revealed statistically significant interactions, sources of significance were ascertained by pairwise post hoc analyses by using the HSD Tukey's test; in all the other cases pairwise comparisons were performed by using two‐tailed *t* tests with Bonferroni's corrected alpha values. Statistical analyses were all carried out with PRISM, GraphPad 8 Software (RRID:SCR_002798; San Diego, USA) with the significance level set at *p* < .05.

## 3 RESULTS

### 3.1 Male RHA and RLA rats display different patterns of sexual behavior that persist after the acquisition of sexual experience

Sexually naïve male RHA and RLA rats exhibited different patterns of copulatory behavior during sexual interaction when put in the presence of a sexually receptive female and these differences were still present, although with some dissimilarities, after the acquisition of sexual experience with five copulatory tests as expected (Sanna et al., 2015, 2019; Sanna, Bratzu, Piludu, et al., 2017; Sanna, Corda, et al., 2014; Sanna, Piludu, et al., 2014). In fact, the two rat lines displayed significant differences in the values of the copulatory parameters either during the first series of the copulatory activity or when considering the entire 60 min of the copulatory test. In particular, both naïve and experienced RHA rats displayed lower latencies to mount, intromit, and ejaculate and higher mount, intromission, and ejaculation frequencies as well as shorter PEI and III and higher CE (Figure 1), and higher TMF, TIF, and TEF in the entire 60 min copulatory test (Figure 2) than their RLA counterparts. Accordingly, two‐way ANOVA detected a significant effect of the rat line on all the parameters considered except MF, a significant effect of sexual experience on all the parameters except MF and IF, and a significant rat line × sexual experience interaction in MF and III. Moreover, pairwise comparisons revealed significant differences between naïve RHA and RLA rats in ML, IL, EL, IF, CE, PEI, III, TIF, and TEF and almost all these differences were still present after five copulatory tests. Finally, sexual experience significantly reduced ML, IL, EL, MF, PEI, and III, and increased CE, TIF, and TEF in RHA rats, reduced ML, IL, and increased TMF and TIF in RLA rats, respectively (see Table 1 for *F* and *p* values from general ANOVAs and Figures 1 and 2 for single points of significance).

### 3.2 Effect of sexual activity on the expression of BDNF, trkB, ΔFosB, and Arc proteins measured by Western blot in the dorsal and ventral hippocampus of sexually naïve and experienced male Roman rats

The antibodies against BDNF, trkB, ΔFosB, and Arc recognized respectively a protein band with a relative MW of ≅13 kDa, in agreement with the reported MW of the monomeric form of the BDNF protein (Rosenthal et al., 1991), a band of ≅140 kDa, consistent with the reported MW of the trkB receptor protein (Klein et al., 1989), a band of ≅36 kDa, consistent with the MW of the short isoform of the FosB protein (Tulchinsky, 2000), and a band of about 55 kDa, in keeping with the expected MW of Arc protein (Lyford et al., 1995) (Figure 3).

As shown in Figure 3, sexual activity induced specific changes in the expression of the markers investigated, depending on the hippocampal subdivision considered (dorsal vs. ventral), on the rat line (RHA vs. RLA) and on the level of sexual experience (control vs. naïve vs. experienced rats). Accordingly, in the dHC (Figure 3a) the assessment of densitometric values by two‐way ANOVA revealed significant effects of the line (for BDNF, trkB, and Arc), level of experience (for BDNF, ΔFosB, and Arc) and a line × level of experience interaction (for Arc) (see Table 2 for *F* and *p* values from general ANOVAs). Moreover, pairwise comparisons revealed that BDNF and Arc were increased in naïve RHA rats compared to their controls (+53% and +176%, respectively) and ΔFosB was increased in experienced compared to both control and naïve rats (+56%, in both cases), while no significant differences due to sexual activity were detected in RLA rats. In addition, significant line differences between RHA and RLA rats were also detected, in particular in the naïve condition for Arc expression (+217% RHA vs. RLA) (see Figure 3a for the significance level of single points).

As regards the vHC (Figure 3b), significant main effects of experience were detected for BDNF and ΔFosB expression (see Table 2 for *F* and *p* values from general ANOVAs). Moreover, pairwise comparisons revealed significant differences between control and experienced RHA rats in ΔFosB expression (+54% in the experienced vs. control rats).

### 3.3 Effect of sexual activity on the expression of BDNF, trkB, c‐Fos, and Arc measured by immunohistochemistry in the dorsal and ventral hippocampus of sexually naïve and experienced male Roman rats

The BDNF‐ (Figure 4a–x, a^1^–r^1^), trkB‐ (Figure 5a–x, a^1^–r^1^), c‐Fos‐ (Figure 6a–x, a^1^–r^1^) and Arc‐like immunoreactivity (LI) (Figure 7a–x, a^1^–r^1^) appeared unevenly distributed within the hippocampal formation. As a rule, the immunoreactive structures looked denser in the RHA than in the RLA rats and in the dHC than in the vHC.

The densitometric analysis in the CA sectors of the hippocampus proper and in the DG (Figures 4, 5, 6, 7) revealed that sexual activity induced specific changes in the expression of the markers investigated depending on the hippocampal subdivision (dorsal vs. ventral), on the hippocampal subregion (CA1, CA2, CA3 sectors of the Ammon's horn and DG), on the rat line (RHA vs. RLA) and on the level of sexual experience (control vs. naïve vs. experienced rats). Accordingly, two‐way ANOVAs detected marker‐ and/or hippocampal subdivision‐ and/or subregion‐dependent significant effects of main factors (i.e., line and level of sexual experience) and interactions (see Table 3 for *F* and *p* values from general ANOVAs).

#### 3.3.1 BDNF

The BDNF‐immunostained structures were mainly represented by nerve fibers and punctate elements distributed among the negative neurons in the thickness of the pyramidal layer of the Ammon's horn, in the alveus and in the dentate gyrus (DG), where they appeared as loose nets and punctate elements distributed in the molecular layer, with increasing density from its outer part to an inner narrow band contiguous to the granular layer, and in the hilus (Figure 4a–x, a^1^–r^1^). Moreover, immunostained neuronal cells, showing labeling in form of intracytoplasmic granules distributed in the perikarya and proximal processes, were found in the pyramidal, molecular, and oriens layers of the Ammon's horn, particularly in the dHC, and in the granular layer, at the interface between the granular and polymorphic layers, and in the hilus of the DG, particularly in the vHC (Figure 4a–x, a^1^–r^1^).

As regards the densitometric analysis, in the dHC line differences between RHA and RLA rats were detected in the whole hippocampal formation and an effect of experience was found in the CA2 and CA3 sectors and in the DG (Figure 4y–b′). Additionally, pairwise comparisons revealed higher BDNF expression levels in naïve RHA rats compared to their controls in the CA3 and the DG (+69% and +63%, respectively); as regards the line differences, a higher BDNF expression was revealed in the CA1, CA2, and CA3 sectors and in the DG of naïve RHA compared to naïve RLA rats (+121%, +168%, +195%, and +151%, in RHA vs. RLA rats, respectively), as well as in the CA1 and CA3 sectors and in the DG of experienced RHA rats compared to their RLA counterparts (+207%, +409%, and +196%, in RHA vs. RLA rats, respectively); in addition, higher BDNF relative levels were found in the CA2 and CA3 sectors and in the DG of control RHA compared to control RLA rats (+403%, +170%, and +128%, in RHA vs. RLA rats, respectively) (see Figure 4y–b ^1^ for the significance level of single points).

In the vHC, the ANOVAs showed an effect of experience in the CA1 and CA3 sectors and in the DG, and a significant effect of line in the CA1 sector. Additionally, pairwise comparisons revealed in the RHA line a significantly higher BDNF expression in the CA3 sector in experienced compared to naïve rats (+51%) and a lower BDNF expression in naïve than in control rats in the DG (−35%) (see Figure 4s ^1^–u^1^ for the significance level of single points).

#### 3.3.2 trkB

The trkB‐labeled structures were also represented mostly by nerve fiber systems, having the aspect of filaments, hollow tubules, and coarse punctate elements (Figure 5a–x, a^1^–r^1^), distributed in between neurons of the pyramidal layer and in the molecular layer of the Ammon's horn, in the alveus, and in between the neuronal cells of the granular layer, in the molecular layer and hilus of the DG (Figure 5a–x, a^1^–r^1^). In the Ammon's horn, trkB‐positive neuronal cell bodies, localized in the pyramidal layer, with labeled proximal processes extending throughout the molecular layer, and in the oriens layer were also observed. In the DG, trkB‐immunostained neuronal perikarya were observed in the hilus (Figure 5a–x, a^1^–r^1^).

As regards the densitometric analysis, in the dHC significant effects of line were detected in all the sectors of the Ammon's horn and a significant effect of experience was found in the DG (see Table 3 for *F* and *p* values from general ANOVAs) (Figure 5y–b′). Additionally, pairwise comparisons revealed significant line differences between controls in the CA2 sector (+71% RHA vs. RLA), between naïve animals in the CA3 sector and the DG (+116% and +46%, in RHA vs. RLA rats, respectively), and between the experienced rats in the CA3 sector (+92% RHA vs. RLA).

In the vHC, only an effect of experience was observed in the DG (see Table 3 for *F* and *p* values from general ANOVAs) and pairwise comparisons revealed in the RHA line a lower trkB expression in naïve rats compared to controls in the DG (−30%) (see Figure 5s ^1^–u^1^ for the significance level of single points).

#### 3.3.3 c‐Fos

The c‐Fos‐immunolabeling was localized to a number of neuronal nuclei in the pyramidal layer of the Ammon's horn and in the granular layer of the DG (Figure 6a–x, a^1^–r^1^).

As regards the densitometric analysis, in the dHC significant effect of experience was detected in all considered subregions while significant effect of line was detected in the CA2 and CA3 sectors and significant line × level of sexual experience interactions in the CA1 and CA3 sectors of the Ammon's horn. Moreover, pairwise comparisons revealed that, in the Ammon's horn, the CA1 sector displayed significant higher expression of c‐Fos in naïve RHA rats compared to both controls and experienced ones (+150% and +899%, respectively), while the CA2 and CA3 sectors of the RHA line, and the CA1 sector of the RLA line showed significant higher expression in naïve than in experienced rats (+285%, +1286%, and +326%, respectively). At variance, in the DG, both Roman rat lines displayed significant higher expression in naïve rats compared to controls and experienced ones (by about +165% and +263%, for RHA and RLA rats, respectively). Finally, significant line‐dependent differences were observed between naïve RHA and RLA rats in all sectors of the Ammon's horn with RHA rats displaying a higher c‐Fos expression than their RLA counterparts (with differences from +79% to +180%) (see Figure 6y–b′ for the significance level of single points).

In the vHC, significant effects of line and experience as well as significant line × experience interactions were detected in the CA1 and CA3 sectors and in the DG (see Table 3 for *F* and *p* values from general ANOVAs) and, similarly to what seen in the dHC, additional pairwise comparisons showed that naïve RHA rats displayed higher expression than both control and experienced animals in the Ammon's horn and DG (with differences from +96% to +803%) as well as a higher c‐Fos expression compared to their RLA counterparts (with differences from +157% to +341%). In contrast, no significant experience‐related differences were observed in RLA rats (see Figure 6s ^1^–u^1^ for the significance level of single points).

#### 3.3.4 Arc

The Arc immunostaining occurred in neuronal perikarya in the Ammon's horn and the DG (Figure 7a–x, a^1^–r^1^).

As regards the densitometric analysis, effects of experience were detected in the CA1 and CA3 sectors and in the DG of the dHC, where an effect of line and a significant interaction were also detected (see Table 3 for *F* and *p* values from general ANOVAs). Moreover, pairwise comparisons revealed higher Arc expression in naïve than in controls and experienced RHA rats in the CA3 and DG (with increases of by about +64% and +300% in the CA3 and DG, respectively) and higher Arc expression in naïve RHA compared to their RLA counterparts in the DG (+225%) (see Figure 7y–b′ for the significance level of single points).

In the vHC, the effect of experience for Arc expression was significant in both the Ammon's horn and the DG (see Table 3 for *F* and *p* values from general ANOVAs); additionally, pairwise comparisons detected lower Arc expression in naïve RHA rats compared to control (−52%) and experienced rats (−53%) in the CA1 sector and to controls in the DG (−52%); similarly, in the RLA line naïve rats displayed lower Arc expression in the CA1 and CA3 sectors when compared to the experienced ones (−49% and −46%, respectively) (see Figure 7s ^1^–u^1^ for the significance level of single points).

## 4 DISCUSSION

To our knowledge, this study shows for the first time that sexual activity (60 min of copulatory activity with a receptive female rat) induces differential changes in the expression levels of putative molecular markers of neural activation, such as c‐Fos and ΔFosB, and of neural plasticity, such as BDNF, trkB, and Arc in the dorsal and ventral subdivisions of the hippocampus of sexually naïve and experienced RHA and RLA rats, when measured either in tissue homogenates using Western Blot or within their sub‐regions, that is, DG, CA1, CA2, and CA3, through densitometric analysis of immunolabeled tissue sections. When present, the changes (increases or decreases) were usually more evident in RHA than in RLA rats, and in particular when rats of both lines were in the naïve rather than in the experienced condition, and in the dorsal rather than in the ventral subdivision of the hippocampus. The differential changes in the expression of BDNF, trkB, c‐Fos, ΔFosB, and Arc in the dHC and vHC of sexually naïve and experienced RHA and RLA rats occured in parallel with the well‐known differences observed in sexual behavior between the two rat lines—RHA rats exhibit higher sexual motivation and better copulatory performances than RLA rats—which are secondary to the higher mesocorticolimbic dopaminergic tone present in RHA versus RLA rats (Sanna et al., 2015, 2019; Sanna, Bratzu, Piludu, et al., 2017; Sanna, Corda, et al., 2014; Sanna, Piludu, et al., 2014).

### 4.1 BDNF/trkB

As extensively shown in Section 3, sexual activity influences BDNF levels in the dHC and vHC and their sub‐regions depending on the rat line, the level of sexual experience, and the hippocampal subdivision considered. The results suggest that sexual activity has opposite effects on BDNF levels in the dHC and vHC and their subregions. Indeed, the BDNF levels measured by Western blot increased in the dHC of both RHA and RLA naïve rats, while they decreased in the vHC of sexually naïve rats of both rat lines compared to control rats. The majority of the above changes are confirmed by immunohistochemistry followed by densitometric analysis. The reasons for these opposite changes are unknown. However, it is reasonable to assume that they reflect the specific functional roles of the two partitions of the hippocampus, the dHC playing the main role in cognitive functions such as learning and memory, and the vHC in anxiety, stress, and affection (Fanselow & Dong, 2010; Tanti et al., 2013; and references therein).

The densitometric analysis also revealed that the opposite changes induced by sexual activity in the dHC and vHC on BDNF expression occur with several differences among the hippocampal subregions in the RHA versus the RLA rats, either in controls or in sexually naïve and experienced rats. In this regard, it is noteworthy that in the dHC, sexual interaction leads in the RHA, but not RLA, rats to an increase of the BDNF expression that occurs mainly in the CA3 sector and the DG; by contrast, a different trend occurs in the vHC, where BDNF is expressed at lower levels in the DG of sexually naïve RHA rats compared to controls and shows higher levels in the CA3 sector of sexually experienced RHA rats compared to their naïve counterparts. The CA3 sector receives the mossy fiber projections, and undergoes a continuous dynamic adjustment of its connectivity, together with processes such as synaptogenesis and dynamic structural plasticity of its pyramidal dendritic arborizations (Leuner & Gould, 2010; Seki & Rutishauser, 1998; Uysal et al., 2012). Thus, it is tempting to speculate that the differential changes in BDNF levels (that occur together with changes in the levels of its trkB receptor, see below) that occur in the dHC and vHC may be related to the differential changes in the adjustments of synaptic connectivity occurring in the dHC and VHC of the two Roman rat lines, which should be expected to be maximal at the first sexual interaction with a receptive female rat and to undergo adaptive changes with the repetition of sexual activity, which leads to the acquisition of sexual experience. This is very evident in RHA rats, but not in RLA rats in which changes in BDNF expression induced by sexual activity are very modest when compared to those observed in RHA rats.

Unfortunately, these findings do not help in clarifying whether the generally lower levels of BDNF‐like immunoreactivity found in RLA rats in this and other studies (Sanna et al., 2019; Serra et al., 2017, 2018) are due to a slower synthesis rate of the BDNF protein or to other causes in this Roman rat line compared to the RHA counterpart. In fact, it is also plausible that the presumably slower synthesis of the BDNF protein in the RLA rats may, in turn, lead to a deficit in the synaptic release and reduced target‐derived support to promote the synaptic contacts with the mossy fibers (Isgor et al., 2015). Nonetheless, this finding suggests that an impaired activity of the BDNF/trkB system might be involved in the different behavioral responses of RHA and RLA when exposed to a sexually receptive female rat (Sanna et al., 2019) or to a 15 min forced swimming test (Serra et al., 2018). The differential changes induced by sexual activity in BDNF levels in the dHC and vHC of RHA versus RLA rats occur together with differences in the levels of trkB, the high‐affinity BDNF receptor (Klein et al., 1989), especially in the dHC, showing in RLA rats trkB levels lower than those of RHA rats. In this regard, it is known that the silencing of trkB receptors in the mPOA decreased consummatory sexual behavior (Brague et al., 2018) and that administration of a trkB antagonist induces lower initial levels of sexual motivation (Hawley & Mosura, 2019).

However, the relation between the differential trkB and BDNF levels in the dHC (and to a lesser extent in the vHC) and the differences in sexual behavior between the two Roman rat lines remain issues to be further investigated, for instance by carrying out experiments involving a direct manipulation of the activity of the trkB receptor and/or of the BDNF‐mediated transmission.

### 4.2 C‐Fos

This study shows that sexual activity also influences c‐Fos levels measured by immunohistochemistry followed by densitometry in the dHC and vHC and their subregions depending on the rat line, the level of sexual experience, and the hippocampal subdivision. Accordingly, c‐Fos levels were increased after sexual activity in the dHC and vHC of both sexually naïve, but not experienced RHA and RLA rats, when compared to control rats, and the higher increases were found in the DG and CA1 subregions followed by the CA2 and CA3 ones of sexually naïve RHA versus RLA rats. However, in spite of the fact that c‐Fos increased in the dHC and vHC of both sexually naïve RHA and RLA rats and that in the DG of the dHC of both RHA and RLA rats c‐Fos levels were very similar, c‐Fos increases were usually higher in both the dHC and vHC in RHA than RLA rats, in which c‐Fos increased only in the dHC. The reason for these differences between sexually naïve RHA and RLA is unknown. Regardless these differences, the similar activation in the DG of dHC in both Roman lines lead to speculate that the first exposition to a sexually receptive female targets discrete cell groups that, independently from the rat line, may represent the hippocampal contribution to reward memory (Gauthier & Tank, 2018; Gava et al., 2021; Schuette et al., 2020). Accordingly, our findings in naïve animals remind the trend of c‐Fos activation upon exposure to environmental novelty (Snyder et al., 2009).

Irrespective of the significance of the differential changes in c‐Fos levels between the Roman lines, the c‐Fos increase after sexual activity in the hippocampus of sexually naïve but not experienced rats resembles the trend of c‐Fos increases that occurs in the VTA, the Acb, and the mPFC of sexually naïve but not experienced RHA and RLA rats (Sanna et al., 2019). These findings suggest that once the sexual experience has been acquired, the raise of c‐Fos levels is not further required for, nor is involved in, the activation of mechanisms facilitating sexual performance and might be secondary to a possible repressive effect of accumulated ΔFosB protein on c‐Fos expression (Renthal et al., 2008; Ruffle, 2014) (see also ΔFosB paragraph below) in experienced animals, as already extensively discussed (Sanna et al., 2019). Although further studies are required to clarify this point, these results show that the hippocampus has to be added to the list of brain areas where c‐Fos levels are increased upon the sexual activity, such as the MPOA, the PVN, the bed nucleus of the stria terminalis (BNST), the medial amygdala, the piriform cortex, the VTA, the Acb, and the mPFC (Beloate et al., 2016; Biały & Kaczmarek, 1996; Bradley & Meisel, 2001; Nishitani et al., 2004; Nutsch et al., 2016; Pitchers, Frohmader, et al., 2010; Veening & Coolen, 1998; Witt & Insel, 1994).

### 4.3 ΔFosB

Sexual activity also influences differentially the levels of ΔFosB, a stable truncated form of the FosB protein that tends to accumulate in brain tissues (Nestler, 2008), in the dHC and vHC of the Roman lines. In fact, ΔFosB increased mainly in both the dHC and vHC of sexually experienced but not naïve RHA rats, with a similar not significant increase tendency in the vHC but not in the dHC of sexually experienced RLA rats. The ΔFosB increase found in the hippocampus of sexually experienced, but not naïve, RHA rats confirms that repeated sexual activity is necessary to increase ΔFosB levels in areas of interest for sexual behavior such as the Acb, VTA, mPFC, mPOA, and caudate‐putamen (Hedges et al., 2009; McHenry et al., 2016; Pitchers, Frohmader, et al., 2010; Sanna et al., 2019; Wallace et al., 2008). These findings are in line with the hypothesis that ΔFosB is a reliable marker of neural activation linked to the acquisition of sexually rewarding experience (Pitchers et al., 2013) and that its expression in the limbic system drives the reward and sexual experience‐induced facilitation of sexual performance (Hedges et al., 2009; Pitchers, Frohmader, et al., 2010; Wallace et al., 2008). However, as found in the VTA, Acb, and mPFC of sexually experienced RLA versus RHA rats (Sanna et al., 2019), the above conclusions are complicated by the very modest ΔFosB increase that occurs in the hippocampus of sexually experienced RLA rats.

The reason why repeated sexual activity increases ΔFosB levels in the hippocampus of RHA much more than in RLA rats is unknown. One explanation may be that the dopaminergic tone generated by VTA neurons that innervate the hippocampus (Gasbarri et al., 1997), which is also very rich in dopaminergic receptors (see Eagle et al., 2015; Kempadoo et al., 2016), is stronger in RHA than RLA rats, as already proposed and extensively discussed for other limbic areas (Acb and mPFC) of the two rat lines. In line with this explanation (i) dopamine from the VTA may regulate the induction of ΔFosB providing a message to hippocampal neurons that relate to the salience and novelty of events (Lisman & Grace, 2005), (ii) dopamine enhances plasticity in CA1 pyramidal neurons (Li et al., 2003) and mediates network‐level activity and memory persistence (McNamara et al., 2014), and (iii) the higher mesocorticolimbic dopaminergic tone of RHA versus RLA rats has been already considered responsible of the higher increase of ΔFosB levels found in the VTA, Acb, and mPFC of sexually experienced RHA versus RLA rats (Sanna et al., 2019). The assumption that the higher levels of ΔFosB in the dHC of RHA versus RLA rats are secondary to a higher dopaminergic tone deriving from the VTA‐hippocampal projection also provides an indication of the still unknown sexual role(s) of ΔFosB expression in the hippocampus. Accordingly, in line with the higher dopaminergic tone of RHA rats and with the recognized role of the dHC in cognitive functions, in particular learning and memory (Fanselow & Dong, 2010), it is reasonable that the higher levels of ΔFosB in the dHC of sexually experienced RHA rats play a role in the faster acquisition of sexual experience by, and/or stabilization of sexual performance in RHA vs. RLA rats. Conversely, since the vHC plays a main role in anxiety, response to stress and emotion (Fanselow & Dong, 2010), and this occurs together with its interaction with the Acb and the mPFC (see Eagle et al., 2020), the differences in ΔFosB levels in this area might play a more general role in the differences in behavioral traits of the two Roman rat lines, including those in sexual behavior (see above), rather than a specific role in the process of acquisition/stabilization of sexual experience. Further studies are required for testing these hypotheses.

### 4.4 Arc

Sexual activity also influences the levels of the Arc protein in the dHC and vHC and their subregions. Accordingly, Arc levels measured by Western Blot increased in the dHC of sexually naïve RHA and RLA rats compared to controls and sexually experienced counterparts. The increases of Arc protein in the dHC were confirmed in the DG and CA3 regions of sexually naïve RHA rats by immunohistochemistry followed by densitometric analysis, which also revealed a significant Arc decrease in the vHC, mainly in the DG and CA1 subregions of sexually naïve RHA rats compared to controls and sexually experienced RHA rats. An Arc decrease tendency was also found in the vHC sub‐regions of sexually naïve RLA rats together with an increase in the CA1 and CA3 sectors of sexually experienced RLA rats compared to sexually naïve counterparts.

Altogether, the obtained results reveal a biphasic pattern of Arc expression within the dHC and vHC during the formation of sexual experience. Indeed, while Arc levels increase in the dHC, they decrease in the vHC of sexually naïve rats after the first copulatory test and return to values similar to controls in sexually experienced rats. Because of Arc role in learning and memory, the increase of its levels in the dHC of sexually naïve RHA rats and, to a lesser extent, of RLA rats, which disappears along with sexual experience in both Roman lines, suggests that the first 60 min of copulatory activity play the crucial role in such increase. Accordingly, this effect disappears after five copulatory tests once the sexual experience is acquired, as found in the VTA, Acb, and mPFC of sexually naïve and experienced RHA and RLA rats (Sanna et al., 2019). In line with this hypothesis Arc is synthesized and then transported at postsynaptic sites where it accumulates to be used when appropriate stimuli linked to the occurrence of new relevant experiences take place (Bramham et al., 2010; Korb & Finkbeiner, 2011; Moga et al., 2004; Shepherd & Bear, 2011), as it may be considered the first exposition to, and copulation with a sexually receptive female rat. Thus, once sexual experience has been acquired and Arc has produced the plastic changes necessary to maintain it, it appears reasonable that Arc content return to its basal (control) values. More difficult is to explain why Arc decreases in the vHC of sexually naïve RHA and RLA rats when compared to controls, a decrease that disappears in sexually experienced rats. Although these findings might be related to the differences in the functional roles of the two hippocampal sub‐divisions (the dHC playing a main role in learning and memory and the vHC in anxiety, stress, and affection, see Fanselow & Dong, 2010), the specific reasons for the observed differential Arc expression in the dHC and vHC after the first sexual intercourse are still unknown. However, since a similar difference occurs also in BDNF expression (BDNF increases in the dHC and decreases in the vHC of sexually naïve RHA and RLA rats, see above), given that BDNF has been found able to regulate Arc activity and expression in neuronal dendrites to produce cytoskeleton rearrangements indispensable for BDNF induced long‐term potentiation in the hippocampus (Cohen et al., 2011; Leal et al., 2015; Messaoudi et al., 2007), it is tempting to speculate that the higher and lower BDNF levels may be involved in the higher and lower Arc levels found in the dHC and vHC of sexually naive RHA and RLA rats, respectively, and that these changes disappear in sexually experienced RHA and RLA rats after the acquisition of sexual experience. Further studies are required to verify this hypothesis.

### 4.5 Final remarks

This study shows that 60 min of copulatory activity induces a series of differential changes in the expression c‐Fos and ΔFosB, Arc, and BDNF/trkB in the dHC and vHC of male RHA and RLA rats, which show different patterns of copulatory behavior (Sanna, Corda, et al., 2014; Sanna, Piludu, et al., 2014). These differences depend on (i) the rat line (RHA vs. RLA); (ii) the level of sexual experience (controls, naïve and experienced); (iii) the hippocampal subdivision and their relevant sub‐regions (dorsal vs. ventral DG, CA1, CA2, and CA3); and (iv) the marker considered. Indeed, when present, the differences were usually more evident in RHA than RLA rats, in the naïve rather than in the experienced condition, and in the dHC rather than in the vHC. The differential changes in BDNF, trkB, c‐Fos, ΔFosB, and Arc levels in the dHC and vHC of sexually naïve and experienced RHA and RLA rats occurred in parallel with the differential changes in sexual behavior of the two rat lines, which are more evident in the naïve condition and tend to decrease but do not disappear in the experienced condition (Sanna, Corda, et al., 2014). These findings confirm that sexual activity induces a neural activation, possibly mediated by c‐Fos and ΔFosB, that leads to trophic and plastic changes mediated by BDNF/trkB and Arc at the level of the dHC and vHC. These changes become stable with repetition of sexual activity, which probably lasts until sexual activity persists. In line with this hypothesis sexual behavior has been found able to induce neurogenesis in the hippocampus of young and middle‐aged male adult rats (Bedos et al., 2018; Glasper & Gould, 2013; Leal‐Galicia et al., 2019; Leuner et al., 2010) and when repeated along time, to stimulate the growth of dendritic spines, leading to a rearrangement of the dendritic architecture in the hippocampus (Leuner et al., 2010), with structural changes that persist until sexual activity is continued in the experimental period (Glasper & Gould, 2013). The results of this study suggest that the stable morphological modifications induced by sexual activity found in the above studies may be secondary to changes in neuronal activation in which c‐Fos and ΔFosB play a key role, and which lead to changes in the trophic and plastic activities of the BDNF/trkB and Arc systems, which occur in the dHC and vHC mainly during the first sexual experience(s). More importantly, the above changes depend on the animal genotypic/phenotypic characteristics, being more evident in RHA than in RLA rats, a finding that is parallel to the higher sexual motivation and better sexual performance of RHA versus RLA rats. Further studies are required to verify whether the sexual activity‐induced differential changes in BDNF, trkB, and Arc levels in the dHC and vHC are causal and/or consequent to the higher sexual motivation and performance of RHA versus RLA rats. Moreover, it is likely that the changes in the expression of BDNF, trkB, c‐Fos, ΔFosB, and Arc between the sexually naïve and the experienced Roman rats that occur in the hippocampus, and in the limbic system in general, may all play a role in the characteristic Roman line differences not only in sexual behavior but also in other motivated behaviors, and their alterations (Fernández‐Teruel et al., 2021; Giorgi et al., 2007, 2019; Melis et al., 2019; Serra et al., 2018).

Finally, this study also confirms that the hippocampus participates in a complex limbic brain circuit connecting areas such as the VTA, Acb, amygdala, and BNST to hypothalamic nuclei (PVN, mPOA) in which neurotransmitters as dopamine and excitatory amino acids, and neuropeptides as oxytocin contribute to mediate the interaction between motivational and consummatory aspects of male sexual behavior (Bratzu et al., 2019; Melis et al., 2007, 2009, 2010; Melis & Argiolas, 2011, 2021a, 2021b; Sanna et al., 2020; Sanna, Bratzu, Argiolas, & Melis, 2017; Succu et al., 2008, 2011). Noteworthy, after sexual activity, differential changes in the expression of BDNF, trkB, c‐Fos, ΔFosB, and Arc, which resemble those found in the hippocampus in this study, also occur in the VTA, Acb and mPFC of sexually naïve and experienced RHA and RLA rats (Sanna et al., 2019).

In conclusion, sexual activity induces differential changes in the expression of BDNF, trkB, c‐Fos, ΔFosB, and Arc proteins in the dHC and vHC of RHA and RLA rats. While the changes in the dHC and vHC are likely to reflect the different specialization of the two hippocampal subdivisions, the more robust protein level changes in RHA versus RLA rats, which occur in particular after the first sexual experience, appear mainly related to the intrinsically different genotype/phenotype of the two Roman rat lines, which leads to differences in the plastic processes at the basis of the acquisition of sexual experience taking place in the hippocampus and other limbic brain areas.

## AUTHOR CONTRIBUTIONS

Fabrizio Sanna, Marina Quartu, Maria Pina Serra, Antonio Argiolas, Maria Rosaria Melis, Osvaldo Giorgi, and Maria Giuseppa Corda designed the project. Maria Pina Serra, Laura Poddighe, Marianna Boi, Antonella Carta, Marina Quartu, and Fabrizio Sanna designed and performed the immunochemical experiments and analyzed the data. Fabrizio Sanna and Jessica Bratzu designed and performed the sexual behavior experiments and analyzed the data. Osvaldo Giorgi, Maria Giuseppa Corda, and Francesco Sanna selected and bred Roman rats. Fabrizio Sanna, Marina Quartu, Antonio Argiolas, Maria Rosaria Melis, Osvaldo Giorgi, and Maria Giuseppa Corda supervised the study and gave interpretation of data. Fabrizio Sanna, Marina Quartu, Antonio Argiolas, and Maria Rosaria Melis wrote the first draft of the manuscript. Fabrizio Sanna, Marina Quartu, Maria Pina Serra, Antonio Argiolas, Maria Rosaria Melis, Osvaldo Giorgi, and Maria Giuseppa Corda read and revised the final version of the manuscript. All authors discussed the results and commented the manuscript. All authors approved the final version of the manuscript.

## CONFLICT OF INTEREST

The authors declare no conflict of interest.

## Data Availability

Data will be made available upon request to the corresponding author.
